# Time to follow up after an abnormal finding in organized gastric cancer screening in Korea

**DOI:** 10.1186/1471-2407-12-400

**Published:** 2012-09-10

**Authors:** Hoo-Yeon Lee, Kui Son Choi, Jae Kwan Jun, Myung-Il Hahm, Eun-Cheol Park

**Affiliations:** 1 Department of Social Medicine, College of Medicine, Dankook University, 201, Manghyang-ro, Dongnam-gu, Cheonan-si, Choongnam, 330-714, Korea; 2 National Cancer Control Institute, National Cancer Center, 111, Ilsan-ro, Ilsandong-gu Goyang-si, Gyeonggi-do, 410-769, Korea; 3 Department of Health Administration and Management, College of Medical Science, Soonchunhyang University, Shinchang-myun, Asan-si, Choongnam, 336-745, Korea; 4 Department of Preventive Medicine & Institute of Health Services Research, College of Medicine, Yonsei University, 50 Yonsei-ro, Seodaemun-gu, Seoul, 120-749, Korea

**Keywords:** Cancer screening, Delays in follow up, Gastric cancer, Organized screening

## Abstract

**Background:**

The prognosis for an abnormal medical finding is affected by both early detection and adherence to the presecribed schedule for follow-up examinations. In this study, we examined the time to follow up after an abnormal finding and determined the risk factors related to delays in follow up in a population-based screening program.

**Methods:**

The study population consisted of patients who were newly diagnosed with gastric cancer through a gastric cancer screening program sponsored by the National Cancer Screening Program (NCSP) in 2005. Due to the skewed nature of the distribution of time to follow up, medians and interquartile ranges (IQR) are presented, and we analyzed the number of days preceding the follow-up time as a binary variable (≤90 days or >90 days). We used logistic regression analyses to evaluate the risk factors for a long delay.

**Results:**

The median number of days to follow-up initiation after an abnormal finding was 11 (IQR 7–27); 13.9% of the patients with gastric cancer obtained their follow-up evaluation more than 90 days. Age, type of health insurance, screening method, and screening results were risk factors for delays in follow up.

**Conclusions:**

This study examined delays from the time of the discovery of an abnormal finding to time of the follow-up evaluation. Because inadequate follow up of abnormal exam results undermines the potential benefits of cancer screening, it is important to organize services that minimize delays between cancer screening and treatment.

## Background

Asian countries, including China, Japan, and Korea, have the highest incidence of gastric cancer in the world [1,2]. Although the incidence of gastric cancer has declined in Korea in recent decades, it remains the most common cancer in this country. Because the prognosis for gastric cancer is favorable when it is detected early, countries with a high prevalence of this disease have sought to reduce the disease burden by providing gastric cancer screening to average-risk populations [3]. In Korea, the National Cancer Screening Program (NCSP), an organized cancer-screening service, recommends biennial gastric cancer screening for males and females older than 40 years of age via either upper gastrointestinal series (UGIS) or endoscopy.

The prognosis for an abnormal medical finding is affected by both early detection and adherence to the presecribed schedule for follow-up examinations. Patient adherence to follow-up recommendations is probably a multifaceted phenomenon [4,5]. Previous research has demonstrated that socioeconomic and demographic characteristics and certain attitudes or misconceptions about cancer are associated with delayed or incomplete follow up. Such attitudes are common among low-income, minority, and underinsured or uninsured populations.

In countries with strong primary healthcare systems, minimizing the time to diagnosis of cancer depends on the ability and willingness of patients to present with potential cancer symptoms and on those of primary care practitioners to respond appropriately to those symptoms either by arranging for further investigation and/or referral to a specialist [6,7]. However, the Korean healthcare system does not have strong gatekeeper system, and the NCSP is not connected with systems that provide further evaluation in the service of diagnosis or treatment. The Korean government provides an opportunity for the population to undergo gastric cancer screening through the NCSP and provides a recommendation for further follow-up evaluation if an abnormality is discovered. Individuals with abnormal findings should then arrange to see a doctor or go to a hospital.

Traditionally, Korean cancer screening programs are evaluated solely on the basis of screening rates. Information about delays in diagnosis or treatment after an abnormal finding on gastric cancer screening tests is scarce. Thus, in this study, we examined the time to follow up after an abnormal finding and determined the risk factors related to delays in follow-up evaluation in a population-based screening program.

## Methods

### National cancer screening program

A nationwide gastric cancer screening program was initiated in Korea in 1999 as part of the NCSP, which recommends biennial gastric cancer screening for males and females older than 40 years of age via either UGIS or endoscopy performed at a clinic, hospital, or general hospital designated as a gastric cancer screening unit by the National Health Insurance Corporation (NHIC).

For those on Medical Aid and in the lower 50% of the NHI premium scale, all such examinations are performed free of charge. People on the upper 50% of the premium scale paid 20% of the screening cost until 2008 and have paid 10% of this cost since 2009. Screening results are notified to all participants within 15 days by letter. Notification process does not differ according to screening results (normal, benign, suspicion of cancer, or cancer). Subjects with findings indicating a suspicion of cancer or cancer are referred for further evaluation or treatment. Participants with abnormal results voluntarily decide whether to make an appointment for consultation with a doctor. Individuals who participate in the NCSP for gastric cancer and are subsequently diagnosed with gastric cancer are also supported financially by the Financial Aid Program for cancer patients. The Financial Aid Program for Cancer Patients was designed to relieve the financial burden of cancer patients. Based on this program, patients suffering from gastric cancers who participated in the NCSP as people on the lower 50% of the premium scale and Medical Aids recipients are financially supported with their diagnosis and treatment. This program covers out of pocket expenses for those medical costs [8].

The overall participation rates in the gastric cancer screening were 17.9% in 2005 and 33.1% in 2008. The positive rates for UGIS and endoscopy were 39.7 and 42.1 per 1,000 screens. The cancer detection rates for UGIS and endoscopy were 0.68 and 2.61 per 1,000 screens. The sensitivities of UGIS and endoscopy for detecting gastric cancer were 36.7 and 69.0%. The specificities of UGIS and endoscopy were 96.1 and 96.0%, respectively [9,10].

### Study population and data sources

We defined the study population as those who had participated in gastric cancer screening through the NCSP in 2005 and who were newly diagnosed with gastric cancer. The sample was selected using the database linking the NCSP database with the Korea National Cancer Incidence Database (KNCIDB). In total, 3083 individuals with complete information were eligible. A total of 692 (28.9%) individuals with a previous diagnosis of gastric cancer according to the KNCIDB were excluded. The final sample consisted of 2,391 subjects.

We used data from the KNCIDB that was collected within 1 year after the initiation of gastric cancer screening in 2005 to enable examination of the diagnostic work-up of information obtained during the 12 months after the first screening and to allow sufficient time for these results to be fully reported. The KNCIDB is a nationwide, hospital-based cancer registry and contains 95% of newly diagnosed malignancies in Korea. We defined a cancer diagnosis according to the criteria of the International Classification of Diseases, code C16 (ICD-10:C16).

The NCSP database includes information on participants’ demographic characteristics, screening date, and screening results. Subjects whose tests were completed were sent a letter that defined the result as “normal,” “benign,” “suspicious,” or “cancer.” We defined results designated as suspicious or indicative of cancer as positive with respect to both UGIS and endoscopy.

We defined the time to follow up as the period from the date of gastric cancer screening to the date of the initiation of follow-up. The date of the initiation of follow-up was defined as the first date when individuals with positive results had consulted with a doctor for their further evaluation and treatment. We analyzed the time to follow up through the linkage between the NCSP data and the data about medical claims obtained from the National Health Insurance Corporation (NHIC). Delays were calculated as number of days. A classification of a long (>90 days ) delay was chosen as the threshold for a follow-up delay. This has been used by other studies, and it is generally assumed that a 3-month delay is too long [6,11-13]. This study was approved by the Institutional Review Board of the National Cancer Center of Korea.

### Data analysis

Our primary outcome was the median number of days from the date of gastric cancer screening to the date of the initiation of follow up. Due to the skewed nature of the data on number of days to follow-up, medians and interquartile ranges (IQR) are presented. We analyzed follow-up days as a binary variable (≤90 days or >90 days) and used the *χ*^2^ test to analyze group differences in the frequencies of categorical variables.

We used logistic regression analyses to evaluate the risk factors for a long delay and included covariates in multivariate analyses. The variables included age, sex, health insurance status as a proxy income indicator, gastric cancer screening method (UGIS, endoscopy), gastric cancer-related “alarm” symptoms (e.g., fatigue, abdominal pain, and discomfort, abdominal bloating, indigestion, changes in bowel habits, and weight loss). The SAS software package (ver. 9.1; SAS Institute Inc., Cary, NC, USA) was used for all statistical calculations.

## Results

### Baseline characteristics of study population

A total of 2,391 individuals who had participated in the NCSP in 2005 were newly diagnosed with gastric cancer (Table 1). Of these, 668 (27.9%) obtained suspicious results, and 1723 (72.1%) were diagnosed with gastric cancer. Of the participants, 401 (16.8%) underwent UGIS and 1990 (83.2%) underwent EGD. The sample included 1641 (68.6%) males and 750 (31.4%) females.

**Table 1:** Distribution of median days to follow-up

**Variables**		**Number (%)**	**Median days**	**IQR**
Total		2391 (100.0)	11	27-7
Age	40–49	360 (15.1)	9	16-6
	50–59	607 (25.4)	11	23-7
	60–69	943 (39.4)	12	28-7
	70+	481 (20.1)	15	48-7
Sex	Male	1641 (68.6)	12	27-7
	Female	750 (31.4)	11	28-6
Result	Suspicion of cancer	668 (27.9)	42.5	308-15
	Cancer	1723 (72.1)	9	15-6
Health Insurance	Medical Aid	145 (6.1)	22	135-6
	NHI premium in the lower50%	954 (39.9)	10	21-6
	NHI premium in the upper50%	1292 (54.0)	12	30-7
Method	UGIS	401 (16.8)	46	302-18
	EGD	1990 (83.2)	10	19-6
Symptom				
Fatigue	No	1981 (82.9)	11	27-7
	Yes	410 (17.1)	12	28-7
Abdominal pain and discomfort	No	1712 (71.6)	12	31-7
	Yes	679 (28.4)	10	20-6
Abdominal bloating	No	2083 (87.1)	11	28-7
	Yes	308 (12.9)	11	22.5-6
Indigestion	No	2104 (88.0)	11	28-7
	Yes	287 (12.0)	11	25-6
Changes in bowel habits	No	2094 (87.6)	11	27-7
	Yes	297 (12.4)	13	28-7
Weight loss	No	2283 (95.5)	12	28-7
	Yes	108 (4.5)	10	18.5-6

### Time to follow-up after an abnormal finding

The median number of days to initiating follow-up after an abnormal finding was 11 (IQR 7-27; Table 1). The median number of days for gastric cancer patients with Medical Aid was 22 (IQR 135–6), compared with 10 days for individuals with NHI in the lower 50% premium scale and 12 days for individuals in the NHI upper 50% premium. The median number of days to follow up was nine among patients with a cancer diagnosis, and it was, 42.5 days for those with suspicious results. The upper IQR among those with suspicious results was 308 days, indicating that 25% of patients did not obtain a follow-up medical evaluation for at least 308 days. Similarly, one-quarter of patients with gastric cancer who were diagnosed via UGIS initiated follow up after 302 days.

According to the results, 59.4% of patients with a suspicious finding from the screening began follow up within 90 days, whereas 96.5% with results indicative of cancer sought follow up within this time frame (Table 2). Patients with gastric cancer who were in their 40s began follow-up within 90 days, compared with 79.2% of those aged 70 or older. Cancer patients with Medical Aid were more likely to have a long delay before follow up than were those with NHI.

**Table 2:** Comparison of study population by follow-up period

**Variables**		**Follow-up time <90 days**	**Follow-up time ≥90 days**	
		**Number (%)**		***P*-value**
Total		2059 (86.1)	332 (13.9)	
Age	40–49	328 (91.1)	32 (8.9)	<0.0001
	50–59	544 (89.6)	63 (10.4)	
	60–69	806 (85.5)	137 (14.5)	
	70+	381 (79.2)	100 (20.8)	
Sex	Male	1418 (86.4)	223 (13.6)	0.5356
	Female	641 (85.5)	109 (14.5)	
Result	Suspicion of cancer	397 (59.4)	271 (40.6)	<0.0001
	Cancer	1662 (96.5)	61 (3.5)	
Health Insurance	Medical Aids	103 (71.0)	42 (29.0)	<0.0001
	NHI premium in the lower 50%	860 (90.2)	94 (9.9)	
	NHI premium in the upper 50%	1096 (84.8)	196 (15.2)	
Method	UGIS	235 (58.6)	166 (41.4)	<0.0001
	EGD	1824 (91.7)	166 (8.3)	
Symptom				
Fatigue	No	1708 (86.2)	273 (13.8)	0.7454
	Yes	351 (85.6)	59 (14.4)	
Abdominal pain and discomfort	No	1449 (84.6)	263 (15.4)	0.0009
	Yes	610 (89.8)	69 (10.2)	
Abdominal bloating	No	1783 (85.6)	300 (14.4)	0.0573
	Yes	276 (89.6)	32 (10.4)	
Indigestion	No	1807 (85.9)	297 (14.1)	0.3774
	Yes	252 (87.8)	35 (12.2)	
Changes in bowel habits	No	1805 (86.2)	289 (13.8)	0.7523
	Yes	254 (85.5)	43 (14.5)	
Weight loss	No	1966 (86.1)	317 (13.9)	0.9991
	Yes	93 (86.1)	15 (13.9)	

### Multivariate analysis

According to the multivariate model, age, screening results, screening methods, health-insurance status, and symptoms of indigestion were associated with follow-up interval (Table 3). Older people had a 0.977 odds ratio (OR) of a follow-up time within 90 days. Participants with screening results indicative of cancer had about a 15 times higher probability of seeking follow up within 90 days than did those with suspicious results. Participants who were screened via endoscopy had about a 1.5 times higher probability of follow up within 90 days compared with those who underwent UGIS. Participants with symptoms of indigestion had about a 1.6 times higher probability of follow up within 90 days than did those without these symptoms.

**Table 3:** Risk factors for delay in follow up after abnormal findings in gastric cancer screening

**Variables**	**OR**	**Upper CI**	**Lower CI**
Age	0.977	0.962	0.991
Sex			
Male	1.000		
Female	0.847	0.630	1.138
Result			
Suspicion of cancer	1.000		
Cancer	14.902	10.606	20.937
Health Insurance			
Medical Aid	1.000		
NHI premium in the lower 50%	2.471	1.368	4.461
NHI premium in the upper 50%	1.516	0.935	2.459
Method			
UGIS	1.000		
Endoscopy	1.481	1.075	2.041
Symptom			
Fatigue			
No	1.000		
Yes	0.900	0.611	1.323
Abdominal pain and discomfort			
No	1.000		
Yes	0.919	0.594	1.423
Abdominal bloating			
No	1.000		
Yes	1.413	0.870	2.295
Indigestion			
No	1.000		
Yes	1.607	1.011	2.555
Changes in bowel habits			
No	1.000		
Yes	1.050	0.681	1.618
Weight loss			
No	1.000		
Yes	0.742	0.377	1.462

## Discussion

In this study, we observed that 13.9% of patients with gastric cancer obtained follow-up evaluation more than 90 days, even though they were told that they needed medical services for diagnostic confirmation and treatment because their screening results were suspicious for cancer or indicated cancer. Age, health-insurance type, screening method, and screening results were risk factors for follow-up delay. Specifically, the 90-day follow-up rate was lower among individuals over 70 years of age, recipients of Medical Aid, individuals with a suspicious results, and those who underwent UGIS compared with individuals who were younger, NHI beneficiaries, individuals with results indicative of cancer, and those who underwent endoscopy, respectively.

These results are consistent with reports from previous studies that cancer screening follow-up delay varies by age and income [4,14-17]. The follow-up delay among individuals aged 70 or over may be due to reluctance to follow the physician’s recommendation to seek diagnostic confirmation or treatment. It may also be more difficult for elderly individuals to understand the need for follow up or to follow up within an appropriate period of time. In the current study, Medical Aid recipients were also less likely than NHI beneficiaries to have an appropriate follow-up period, even though recipients of Medical Aid who participated in the NCSP for gastric cancer and was subsequently diagnosed with gastric cancer through the program would be supported financially through the Financial Aid Program. This indicates financial or other barriers may affect recipients of Medical Aid despite free-of-charge cancer screening and the Financial Aid Program for treatment. These people may be dealing with other issues, including difficulty in accessing facilities and multiple personal and cultural barriers to choosing to undergo follow-up evaluations [18]. Participants who underwent endoscopy may have responded more rapidly to abnormal results because they believed endoscopy to be more accurate than UGIS in detecting gastric cancer [18].

The literature contains no consensus about the definition of a “reasonable” follow-up interval after an abnormal screening result for gastric cancer. Some investigators have found that follow-up intervals of up to 3 months may not impact overall survival from breast or colorectal cancer, whereas others have shown that women who waited more than 30 days for evaluation after the detection of breast cancer were more likely to experience cancer recurrence or death [4,14,19]. The general perception among patients, health professionals, and politicians is that delay has a definitely unfavorable effect on outcome [7,11,12,20-22].

The cancer-care-continuum disparities model begins with prevention and early detection and continues through the survival period. Several of the factors that may contribute to cancer disparities may occur at each end of the continuum or at the stages in between, such as diagnosis and treatment [14,18,23,24]. This study was unable to address the long-term clinical significance of the delay in follow up after an abnormal gastric cancer screening result; however, similar differences at other points along the cancer-care continuum may have a cumulative clinically significant overall impact on mortality. Inadequate follow up of abnormal exam results undermines the potential benefits of cancer screening [25]. An organized cancer screening program is one of many interventions for reducing disparities in the cancer-care continuum. Services from cancer screening to treatment need to be organized so that delays are minimized. Improvements in the diagnostic and treatment process should be achievable, particularly for those whose delays were longest. The design of tailored and targeted interventions such as campaigns directed at specific age groups and social classes may help to reduce these delays. Interventions should involve the local community and should be related to each aspect of the aforementioned barriers that may contribute to delays in follow-up.

Several European countries with strong primary healthcare systems, such as the United Kingdom and Denmark, have developed organized cancer screening systems to reduce the time for the diagnosis and treatment of cancer. National fast-track referral guidance has also recently been introduced. Suspicion of cancer leads to prompt referral of the patient to a specialist for assessment and initiation of a progressive diagnostic program that is conducted within a limited time frame [11].

In the context of a gastric cancer screening unit, the absence of the sort of close relationship or continuous connection that exists between primary physicians and patients may have affected the long follow-up periods found by the current study. The use of primary-physician-based intervention may be a good way to decrease the follow-up time. Additionally, the Public Health Centers in Korea are now primarily responsible for encouraging target individuals to participate in the NCSP. The Public Health Centers need to encourage the public, via telephone or letter, not only to participate, but also to follow up and maintain an appropriate follow-up timeline after an abnormal finding by campaigning. The NCSP needs to develop a quality improvement (QI) infrastructure to implement an effective strategy for improving follow up [26].

Follow-up delay may be affected by knowledge of a malignancy, perceptions of the results as reported in the letter, or access to services [6,7]. Sensitive and specific strategies to reduce patient delays, especially those related to knowledge of a malignancy or perceptions of the letter reporting the screening results, should improve awareness of cancer and aid in patients’ interpretation of screening results. Delays may be reduced by crafting letters that present the results in a way that is easily understood by underprivileged participants according to age, education, and income; that provides clear referral guidance or a referral protocol; or that enables access to a well-organized system that facilitates prompt action by participants with abnormal findings.

This study has several limitations. First, we did not have information about beliefs about screening, health literacy, patient–provider communication and relationships, or system failures. It would be useful to collect such information in future studies to enable a more comprehensive analysis of our findings. Second, we had no information about participants who did not use medical services at all. Work is needed to further explore the reasons that patients do not see a doctor at all and to examine the implications of this behavior. Finally, information about stage distribution was not available. It would be informative to compare stage distribution between those who had follow-up delay or not.

Some recent studies in Korea have focused on participation in cancer screening programs, especially organized cancer screening programs [27-33]. One study suggested that progress had been made toward bridging disparities in organized screening for gastric cancer that were related to socioeconomic status [34]. However, policy makers have paid little attention to delays or variations in the initiation and completion of treatment until now. This study provides additional evidence of follow-up delay and variations in the time to follow up after an abnormal finding. This information may be used in subsequent analyses to identify barriers that lead to differences in the lengths of such delays. Interventions can then be implemented to address delays between the diagnosis and treatment of gastric cancer.

## Conclusions

This study provides additional evidence of follow-up delay and variations in the time to follow up after an abnormal finding. An organized cancer screening program is one of many interventions for reducing disparities in the cancer-care continuum. Inadequate follow up of abnormal exam results undermines the potential benefits of cancer screening. Therefore, it is important to organize services that minimize delays and decrease variations in the time between cancer screening and treatment.

## Competing interests

The authors declare that they have no competing interests.

## Authors’ contributions

HYL participated in the design of the study, analyzed and drafted the manuscript. KSC, JKJ, and MIH helped to perform statistical analysis and data interpretation. ECP participated in study design and interpretation, and critically revised the manuscript. All authors read and approved the final manuscript.

## Pre-publication history

The pre-publication history for this paper can be accessed here:

http://www.biomedcentral.com/1471-2407/12/400/prepub
